# A survey on practitioners’ attitudes toward artificial intelligence in radiology

**DOI:** 10.3325/cmj.2023.64.289

**Published:** 2023-08

**Authors:** Tin Orešković, Žiga Snoj, Mili Sanwalka, Boris Brkljačić, Mirjana Kujundžić Tiljak, Stjepan Orešković, Ivo Dumić-Čule

**Affiliations:** 1 University of Oxford, Big Data Institute, Oxford, United Kingdom tin.oreskovic@balliol.ox.ac.uk; 2 Ljubljana University Medical Center, Ljubljana, Slovenia; 3 Harvard Business School, Boston, MA, USA; 4 Department of Diagnostic and Interventional Radiology, University Hospital Dubrava, Zagreb, Croatia; 5 University of Zagreb School of Medicine, Zagreb, Croatia; 6 University of Zagreb School of Medicine, Andrija Štampar School of Public Health, Zagreb, Croatia; 7 University North, Varaždin, Croatia; **Orešković et al:** A survey on practitioners’ attitudes towards artificial intelligence in radiology

The perception of functionality and reliability, and appropriate levels of expert supervision of artificial intelligence (AI) tools are key factors determining their adoption in everyday radiology practice. Research on this topic has mainly focused on general awareness about AI, radiologists’ familiarity with AI tools, and their expectations regarding the future of the profession (1-3). Expanding on this literature, we conducted a survey to assess attitudes toward AI tools among radiologists and radiology residents in two European Union (EU) countries: Croatia and Slovenia.

We distributed an anonymous questionnaire among radiologists and radiology residents in the two countries. The respondents were practicing in primary, secondary, and tertiary health care institutions, both in the private and public sectors. We assessed the agreement with nine statements about reliability, trustworthiness, and appropriate levels of expert supervision with responses on the Likert scale (from strongly disagree to strongly agree). We analyzed the responses statistically by dichotomizing them into those expressing agreement or strong agreement with the question/statement and all other responses (including the “no stance” response). The responses, assigned values between 1 (strongly disagree) and 5 (strongly agree), were additionally combined into a single “confidence in AI” mean score (with scoring for negative statements inverted) in order to simplify testing associations with age group, usage of AI tools in everyday practice, and the area of subspecialization.

**Figure 1:**
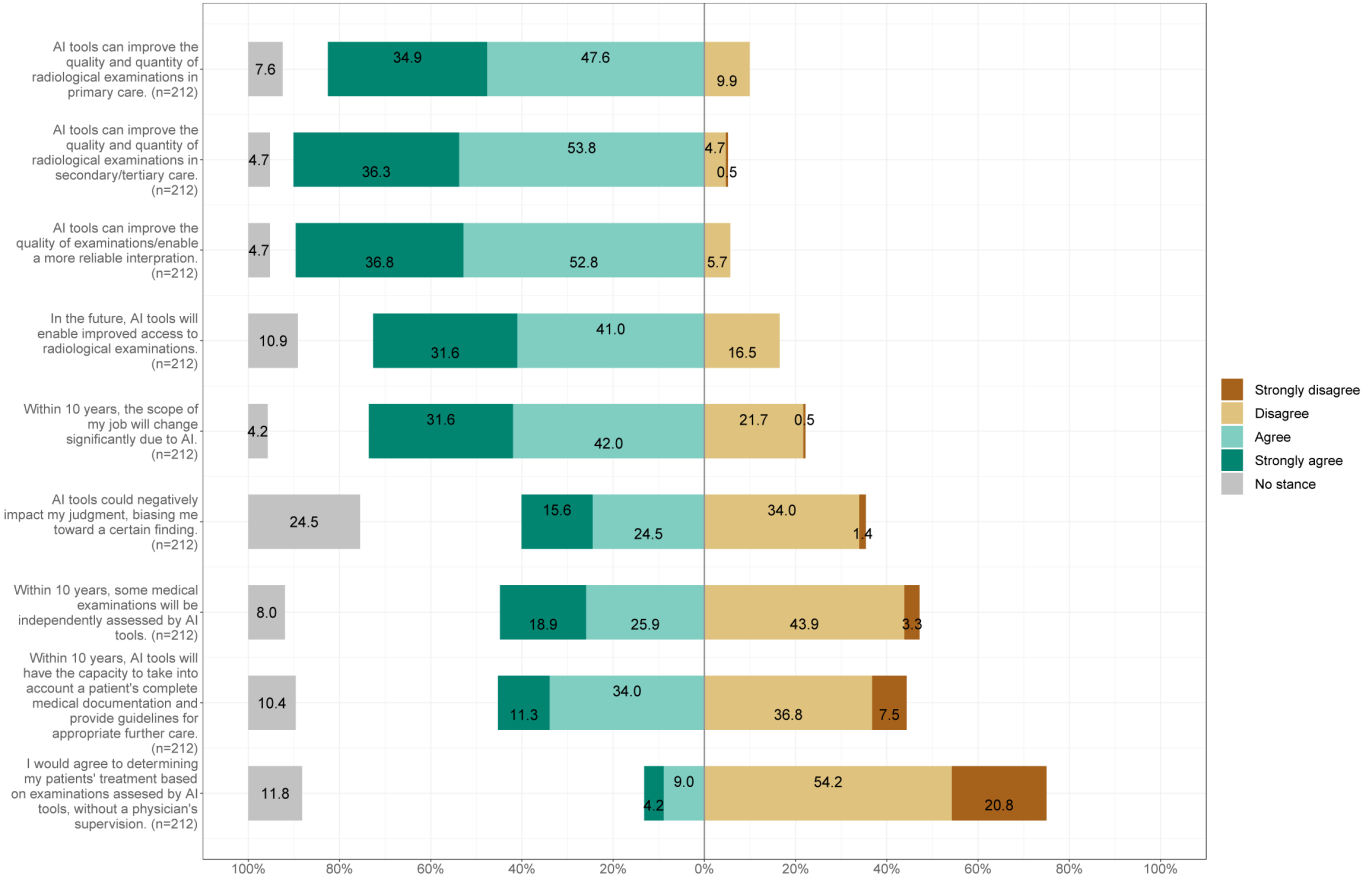
Agreement with nine statements on the use of artificial intelligence (AI) tools in radiology.

The overall response rate was 34% (212 of 631). Twenty-nine respondents (14%) reported using AI tools in their everyday work. Figure 1 reports the frequencies of each response for each of the nine statements, while Table 1 presents the dichotomized mean responses and 95% confidence intervals. More than 70% of respondents believed AI could improve the quality of radiological examinations, enable their more reliable interpretation, and improve access to this type of examinations; more than 70% also expected the scope of their job to significantly change within ten years. Forty-five percent expected that AI tools would, within ten years, have the capacity to take into account patients’ complete medical documentation and provide appropriate guidelines for care, and that some examinations would be assessed by AI tools independently of human experts. Forty percent believed AI tools could negatively impact their judgment, while 13% stated they would agree to determine a patient’s treatment based on an AI tool’s recommendations without supervision. Finally, the mean “confidence in AI” score was 3.5 out of 5 (standard deviation: 0.55). Table 2 presents the associations of the score with age group (compared with age <30), an indicator for working with AI tools every day, and area of subspecialization (in comparison with no subspecialization).

**Table 1:** Mean dichotomized agreement with nine statements on artificial intelligence (AI) tools in radiology

Positive responses (/212)	Mean	95% confidence interval	Question
175	0.83	0.77-0.87	AI tools can improve the quality and quantity of radiological examinations in primary care.
191	0.90	0.85-0.93	AI tools can improve the quality and quantity of radiological examinations in secondary/tertiary care.
190	0.90	0.85-0.93	AI tools can improve the quality of examinations/enable a more reliable interpretation.
154	0.73	0.66-0.78	In the future, AI tools will enable improved access to radiological examinations.
156	0.74	0.67-0.79	Within 10 years, the scope of my job will change significantly due to AI.
85	0.40	0.34-0.47	AI tools could negatively impact my judgment, biasing me toward a certain finding.
95	0.45	0.38-0.52	Within 10 years, some medical examinations will be independently assessed by AI tools.
96	0.45	0.39-0.52	Within 10 years, AI tools will have the capacity to take into account a patient's complete medical documentation and provide guidelines for appropriate further care.
28	0.13	0.09-0.18	I would agree to determining my patients' treatment based on examinations assesed by AI tools, without a physician's supervision.

**Table 2:** Linear regression of confidence in artificial intelligence (AI) score (range 0 to 5) on age, subspecialization, and the use of AI tools in everyday work

Variable	Coefficient	95% confidence interval
Intercept	3.34	3.09-3.59
Age group:		
30-39	0.12	-0.1-0.33
40-49	0.26	0.01-0.52
50-59	0.18	-0.13-0.5
60-69	0.47	-0.03-0.96
Subspecialization:		
abdomen	-0.07	-0.32-0.17
breast	-0.06	-0.35-0.23
head and neck	-0.26	-0.94-0.41
interventional radiology	0.02	-0.29-0.34
musculoskeletal system	0.27	-0.01-0.54
neuroradiology	-0.16	-0.43-0.11
thorax	-0.03	-0.33-0.27
Uses AI tools in everyday work	0.13	-0.09-0.35

Despite limited exposure to AI tools in practice and standard medical education (4,5), and in contrast with the earlier results of a study by Jungman et al (6), responses were on average highly positive to statements about the reliability and current functionalities of AI tools, as well as expectations concerning their future use. Expectations of and support for the use of AI tools without human oversight were expectedly lower. A considerable minority (40%) of respondents also expressed worry that AI tools could negatively affect their judgment. These attitudes did not vary substantially with age, the use of AI tools, or the area of subspecialization. Overall, the survey showed high confidence levels in AI tools, indicating a professional climate receptive to greater integration of such tools in practice.
